# METTL3 Inhibition Suppresses Cell Growth and Survival in Colorectal Cancer via ASNS Downregulation

**DOI:** 10.7150/jca.96760

**Published:** 2024-07-16

**Authors:** Yang Yu, Yanan Hai, Hongfeng Zhou, Wenfang Bao, Xiaowei Hu, Yong Gao, Jin Wu

**Affiliations:** 1 Harbin Medical University Cancer Hospital, Harbin 150081, Heilongjiang Province, China.; 2 Department of Oncology, Shanghai East Hospital, Tongji University School of Medicine, Shanghai 200120, China.

**Keywords:** STM2457, ASNS, METTL3, colorectal cancer, m6A modification

## Abstract

**Background:** Colorectal cancer (CRC) presents a significant global health burden, with high rates of incidence and mortality, and an urgent need to improve prognosis. STM2457, a novel small molecule inhibitor specific for N6-methyladenosine (m6A) catalytic enzyme Methyltransferase-like 3 (METTL3) has implicated significant treatment potentials in a few of types of cancer. However, its impact and underlying mechanism are still unclear in CRC cells.

**Methods:** We used CCK-8 and colony formation assay to observe cell growth, flow cytometry and TUNEL approaches to detect cell apoptosis under the treatment of STM2457 on CRC cells* in vitro* or *in vivo*. RNA-sequencing, qRT-PCR and western blotting were performed to explore downstream effectors of STM2457. Messenger RNA stability was evaluated by qRT-PCR after treatment with actinomycin D. The methylated RNA immunoprecipitation (MeRIP) qPCR, dual-luciferase reporter analyses and m6A dot blotting were carried out to measure the m6A modification. Associated gene expression pattern and clinical relevance in CRC clinical tissue samples were analyzed using online database.

**Results:** STM2457 exhibited a strong influence on cell growth suppression and apoptosis of CRC cells *in vitro* and subcutaneous xenograft growth *in vivo*. Asparagine synthetase (ASNS) was markedly downregulated upon STM2457 treatment or METTL3 knockdown and exogenous overexpression of ASNS could rescue the biological defects induced by STM2457. Mechanistically, the downregulation of ASNS by STM2457 may be due to the decrease of m6A modification level in ASNS mRNA mediated by METTL3.

**Conclusions:** Our findings suggest that STM2457 may serve as a potential therapeutic agent and ASNS may be a new promising therapeutic target for CRC.

## Introduction

Colorectal cancer (CRC) represents a significant health challenge globally over the last three decades 1. Statistically, CRC ranks third among new cancer cases and second among cancer-related deaths worldwide 2,3. In addition, by 2030, the incidence rate of CRC will increase by over 2.2 million, resulting in more than 1.1 million deaths 4. The prognosis of CRC patients remains unsatisfactory despite the application of a range of advanced therapeutic approaches, such as surgical excision, chemotherapy, targeted therapy, radiotherapy and immunotherapy 5,6. Accordingly, it is imperative to develop diverse and effective novel treatment for CRC patients.

Growing evidence indicates that RNA modification pathways are responsible for cancer progression and can be well-targeted for the cancer treatment 7. Ribonucleic acid (RNA) N6-methyladnosine (m6A) is the most general and plentiful RNA modification 8. The modification process of m6A in mammals is dynamically reversible and regulated by, "erasers", and "readers". Among these factors, research on “writers” has been very extensive and continuously improving 9,12. At present, the m6A methyltransferase complex (MTC) as a main of the "writers", consisting of METTL3/METTL14 and other components, was discovered as the first m6A methyltransferase 13. Especially, of the complex, METTL3 mainly acts as the catalytic core, while METTL14 and others serve as regulatory subunits 14. During recent years, there have been various studies on m6A mediated by METTL3. With the research on the regulation, function, and mechanism of METTL3 in cancer, multiple pathways of cancer affected by METTL3 have been discovered, mainly focusing on cell proliferation, cell death resistance, invasion and metastasis, angiogenesis, metabolic disorders, and immune 41,42.

In recent years, numerous studies have demonstrated the crucial role that METTL3 plays in the progression of CRC. Currently, mainstream studies have confirmed the high expression of METTL3 in CRC, and METTL3 is closely related to tumor immune escape, glucose metabolism and drug resistance of CRC 15,16,17,18. Targeted treatment of METTL3 may be an effective individualized therapeutic approach for this disease. STM2457, a highly efficient and selective catalytic inhibitor of METTL3, has been proven to successfully prevent the proliferation and progression of AML *in vitro* and *in vivo* for the first time 19. Since then, several cancer studies related to METTL3 have also used STM2457 and validated its efficacy in inhibiting tumor progression 20,21. To date, comprehensive research on the effects of STM2457 on CRC is still limited. To this goal, we have designed various cellular and molecular biological experiments to explore the effects and mechanisms of STM2457 on CRC, with the expectation of providing a promising and effective therapeutic strategy for CRC.

## Materials and methods

### Chemicals and antibodies

STM2457 (HY-134836) was acquired from MedChemExpress, Ltd (USA) and dissolved in dimethyl sulfoxide (DMSO). Primary antibodies ASNS (1:1000, 14681-1-AP), β-Actin (1:3000, 23660-1-AP) and METTL3 (1:1000, 14681-1-AP) were obtained from Proteintech, Wuhan, China.

### Cell lines and cell culture

HCT116 and SW620, the two kinds of human CRC cell lines, were obtained from Shanghai Cell Bank of Chinese Academy of Sciences. HCT116 and the SW620 cells were cultured in Dulbecco's Modified Eagle's Medium (DMEM, Sigma, Germany) supplemented with 10% fetal bovine serum (FBS, Corning, USA) and 1% Penicillin-Streptomycin (P/S, Gibco, USA). Cells were grown in 95% humidity with 5% CO_2_ at 37℃.

### RNA-sequencing

CRC cell line HCT116 was treated with DMEM containing DMSO or STM2457 (20μM) for 48 h. In a word, the total RNA of cells isolated using TRIzol reagent was immediately rapidly frozen in liquid nitrogen. After passing the total RNA quality control, poly (A) mRNA separated from beads with oligo (dT) and then divided into small fragments at high temperature. Then create a cDNA library using reverse transcription cleaved RNA fragments. RNA-sequencing was performed on the NovaSeq6000 platform and raw data were achieved. Raw data were processed and filtered 24. The obtained pure reads are mapped to the human genome (GRCh38) and further processed for statistics and counting. Differential expression analysis was performed using the edgeR package. The genes in conditions of p<0.05 and |log2FC|>1 were supposed to be differentially expressed. The raw data of RNA sequencing in this paper is available in the SRA (Sequence Read Archive) database with accession number PRJNA1111514.

### Colony formation and Cell Counting Kit-8 assays

Colony formation assay, cells were placed and spread evenly in a six-well plate at a density of 1,000. After 2 weeks of cultivation, the cells were fixed with 4% paraformaldehyde for 5 minutes and stained with 0.5% crystal violet solution for 20 minutes. And the Cell Proliferation assays, the Cell Counting Kit-8 reagent (CCK-8, DoJindo Laboratories, Japan) was adopted for analyzing cell growing trend. To put it simply, the cells were seeded into a 96-well plate at a density of 3 × 10^3^ cells per well in triplicate. 0μM, 20μM and 40μM concentration of the drug were added to treat CRC cells, and 10μl of CCK8 reagent was added to the wells to be measured at a fixed time point for 5 consecutive days, incubated for 70 minutes. The last step, the absorbance at 450 nm wavelength was measured by a spectrophotometer. The above experiments were repeated no less than three-time.

### Cell apoptosis analysis

CRC cells were cultured in DMEM containing different concentrations of STM2457 (0,20,40 μ M) for 48 hours. According to the instructions, the cells were collected separately and resuspended with 1×Annexin V Binding Solution, then FITC and PI solutions were added to the cell suspension. Subsequently, the suspension was placed in dark and at room temperature for 15 minutes, and flow cytometry was applied to measure cell apoptosis. The reagent used in cell apoptosis experiment is Annexin V, FITC apoptosis detection kit (DoJinDo Laboratories, Japan). The obtained results were analyzed by FlowJo software (FlowJo, LLC). All experiments were repeated at least three times.

### Western blot analysis

Adopting 1 × RIPA buffer (#20-188, Merck Millipore, USA) was mixed to the inhibitor cocktail (Roche, Indianapolis, IN) to prepare a mixture solution. Placed the CRC cell culture dish on ice and sucked out DMEM. The mixture solution was added to the dish to lyse the cells for 15 minutes. And then in a 4 ℃ centrifuge for 15 minutes. Diluted the cell lysate by adding 5 × SDS loading buffer, mix well, then boil for 10 minutes. The obtained cell proteins were separated using SDS-PAGE and electroimprinted onto a nitrocellulose filter membrane. Add 5% non-fat dry milk to PBST (PBS containing 1% Tween-20) and place the membrane in the mixed solution at room temperature for blocking 1 hour. Next, the membrane was incubated with the specific first antibody at 4 ℃ overnight. The membrane was washed three times on the shaker with PBS containing 0.05% Tween-20, each for 5 minutes, afterwards, incubating with the corresponding secondary antibody for 1 hour and washing the membrane once more though the same method. Visualize protein bands using the Odyssey Infrared Imaging System (LI-COR Biosciences).

### RNA isolation, reverse transcription and real-time quantitative PCR (qRT-PCR)

Total RNA was extracted from CRC cells with the TRIzol reagent based on the manufacturer's recommendations. Use 1 μg total RNA for reversing transcription in 20 μl reaction system by PrimeScript RT reagent kit (Takara, Japan). The cDNA was analyzed by qPCR accomplished with TB Green Premix ExTaq™ kit (Takara, Japan) in a QuantStudio™ 6 Flex Real-Time PCR System. Calculated the relative expression levels of target genes through the 2-ΔΔCt method, and human β-actin was used as the internal control. Each sample was repeated three times at the least. The primers used in the experiment were as follows:

ASNS-Forward: 5′-GGAAGACAGCCCCGATTTACT-3′,

ASNS-Reverse: 5′-AGCACGAACTGTTGTAATGTCA-3′,

METTL3-Forward: 5′-AGATGGGGTAGAAAGCCTCCT-3′,

METTL3-Reverse: 5′-TGGTCAGCATAGGTTACAAGAGT-3′,

β-actin-Forward: 5'-AGAGCCTCGCCTTTGCCGATCC-3',

β-actin-Reverse: 5'-CTGGGCCTCGTCGCCCACATA-3'.

### Dual-luciferase reporter assay

First of all, a wild-type and a mutant (mutation of base A at positions 2257 into T) 3'-UTR fragment of ASNS were synthetized and inserted into luciferase reporter pGL3 basic vector (Sangon Biotech, Shanghai, China). Subsequently, 5×10^3^ CRC cells were placed in the 96-well plate and were co-transfected with 150 ng of empty, pGL3-ASNS-WT or pGL3-ASNS-MUT and 2ng of pRL-TK (Promega, Madison, WI, USA) with the CRC cells by using Lipofectamine 3000 (Invitrogen, Carlsbad, California, USA) for 48 hours. According to the instructions we analyzed the luciferase activity applying a Dual-Luciferase ® Reporter (DLR™) Assay System (Promega, USA). The activity ratio between renal luciferase and firefly luciferase was used for measuring the relative activity of luciferase.

### MeRIP-qPCR

The methylated RNA immunoprecipitation (MeRIP) assay procedure was performed in accordance with instruction manual of the manufacturer using a Magna MeRIP™ m6A kit (#17-10499, Millipore, Germany). In brief, total RNA was extracted first of all and 5ul was separated as input. Premixed A/G immunomagnetic beads and m6A antibodies according to the standard steps of the kit. Add the premixed solution to RNA and incubate them together at 4 ° C for 4 hours. After adequate washing, the immunoprecipitate was digested by protease K, and then RNA was extracted. Finally, qPCR was carried out together with input RNA. The primer pair used as follows: Forward: 5′-CGCTGACCCACTACAAGTCA -3′, and Reverse: 5′-TTAGCCTGAGTTGACTCTCA-3′.

### Transfection and lentiviral transduction for stable cell lines

The lentiviruses of overexpressing ASNS, overexpressing METTL3, and knocking down METTL3 (including shNC and empty vector lentivirus) were all purchased from GenePharma (Shanghai, China). We seeded the CRC cells into plates and used Polybrene (5μg/μl) to transfect them with above lentiviruses. In order to establish stable cell lines, CRC cells transfected with these lentiviruses were screened with puromycin (2 µg/ml, S7417, Selleck) for at least one week. We mutated the key catalytic active sites (D395A and W398A) of the METTL3 plasmid (GenePharma, Shanghai, China). Thereafter we transfected the wild-type and mutant plasmids of METTL3 and empty-vector into cells with Lipofectamine 3000 for 24 hours.

### HE staining, immunohistochemistry and TUNEL assay

Mouse tumor tissue was fixed overnight with 4% paraformaldehyde. Afterwards, the tissue we obtained were dehydrated and embedded in paraffin. The wax blocks were then cut into 5µm sections. We stained the sections with hematoxylin and eosin. For the immunohistochemical staining, the wax slices were dewaxed and hydrated before cleaning, and then the slices were subjected to antigen thermal repair using citrate buffer solution. Incubate the sections with Ki67 (ab15580, Abcam Trading Ltd, England) rabbit polyclonal antibodies at 4 ° C for 12 hours. Subsequently, the sections are washed and incubated with secondary antibodies. Finally, the sections are re-stained with hematoxylin staining solution. The Tunel Assay was completed in accordance with the instructions provided by the reagent manufacturer (C1091, Beyotime Biotech Ltd, China). In a few words, the slices were incubated with protease K after paraffin removal. Thereafter, the slices were washed and incubated at room temperature in 3% H_2_O_2_ solution prepared with PBS for 20 minutes. The slices were washed again and added with the biotin labeling solution. Then, the slices were incubated with Streptavidin-HRP and DAB chromogenic solution and finally stained with hematoxylin.

### RNA m^6^A dot blotting assay

Used TRIzol reagent to extract total RNA from the test samples according to the method described above. The same group of RNA was diluted to 800ng/ul and 400ng/ul respectively. After 1ul RNA liquid droplets were placed on nitrocellulose membranes, RNA crosslinking was carried out under ultraviolet light. Seal the membrane with skim milk (5%) for 1 hour. Then incubated with m6A antibody (1:1000, #56593S, Cell Signaling Technology, Boston, USA) overnight at 4 ℃. Washed the membranes with PBST. Subsequently incubated with Anti-rabbit IgG HRP-linked antibody (1:3000, # 7074 MA Cell Signaling Technology, Boston, USA) for 1 hour at room temperature. After washing the membranes with PBST, chemiluminescence development was performed. Dropped the same sample of 1ul on other nitrocellulose membranes, cross-link under ultraviolet light, dyed with 0.02% methylene blue for 30 minutes and washed with ddH_2_O for 15 minutes as quantitative control of RNA.

### RNA stability assay

After CRC cells were treated with DMSO or STM2457 (40 μM) for 24 hours, the new DMEM was replaced and 5 μg/ml actinomycin D was added. Then the cells were collected and total RNA was extracted at 0 h, 3 h and 6 h after adding actinomycin D. The total RNA was analyzed by qRT-PCR to analyze the mRNA level using the method mentioned above.

### Source and processing of bioinformatics analysis

We analyzed the expression of ASNS in CRC using cancer genome data sharing platform The Cancer Genome Atlas (TCGA) with online analysis tool Gene Expression Profiling Interactive Analysis (GEPIA). We also studied the correlation between METTL3 and ASNS at the mRNA level through GEPIA. We used Kaplan-Meier plotter to evaluate the prognosis of patients with different levels of ASNS expression.

### Animal experiments

We purchased athymic nude mice which were 4-5 weeks old from Gempharmatech Ltd (Nanjing, China) and established the xenograft models of CRC. For the first group of animal experiments, ten mice were injected subcutaneously with an equivalent amount of HCT116 cells (2×10^6^ cells) under the armpit area, and then randomly divided into two groups (n=5). All mice were treated with vehicle or STM2457 (50 mg/kg, every three days, intraperitoneal injection) at the same dose consecutively after two weeks. In the same way, the SW620 xenograft mouse model was constructed and administered. For the second group of animal experiment, equal quantity HCT116/VEC and HCT116/ASNS cells (2×10^6^ cells) were injected into each side of armpit of the nude mouse subcutaneously, individually, randomly divided into 2 groups (n=5). All mice were treated in the same manner and dosage as the first group. Regularly measure tumor volume every 3 days throughout the entire treatment process. After 2 weeks of treatment, the mice were sacrificed. Eventually, we measured the tumor weight and volume. The formula for calculating tumor volume: length×width^2)/2. All the animal experiments were performed under protocols approved by the Animal Care and Use Committee of Shanghai East Hospital, Tongji University.

### Statistical analysis

All data are expressed as mean ±standard deviation (SD). The two-tailed Student t-test or one-way analysis of variance (ANOVA) was utilized for compared between groups. Statistical analysis was implemented by GraphPad Prism 8 (GraphPad Software, Inc.). Statistical significance was noted as P values < 0.05, and the different levels of significance were set to p<0.05 (*) and p<0.01 (**).

## Results

### STM2457 has anti-cancer impacts on CRC cells *in vitro*

**Figure 1:**
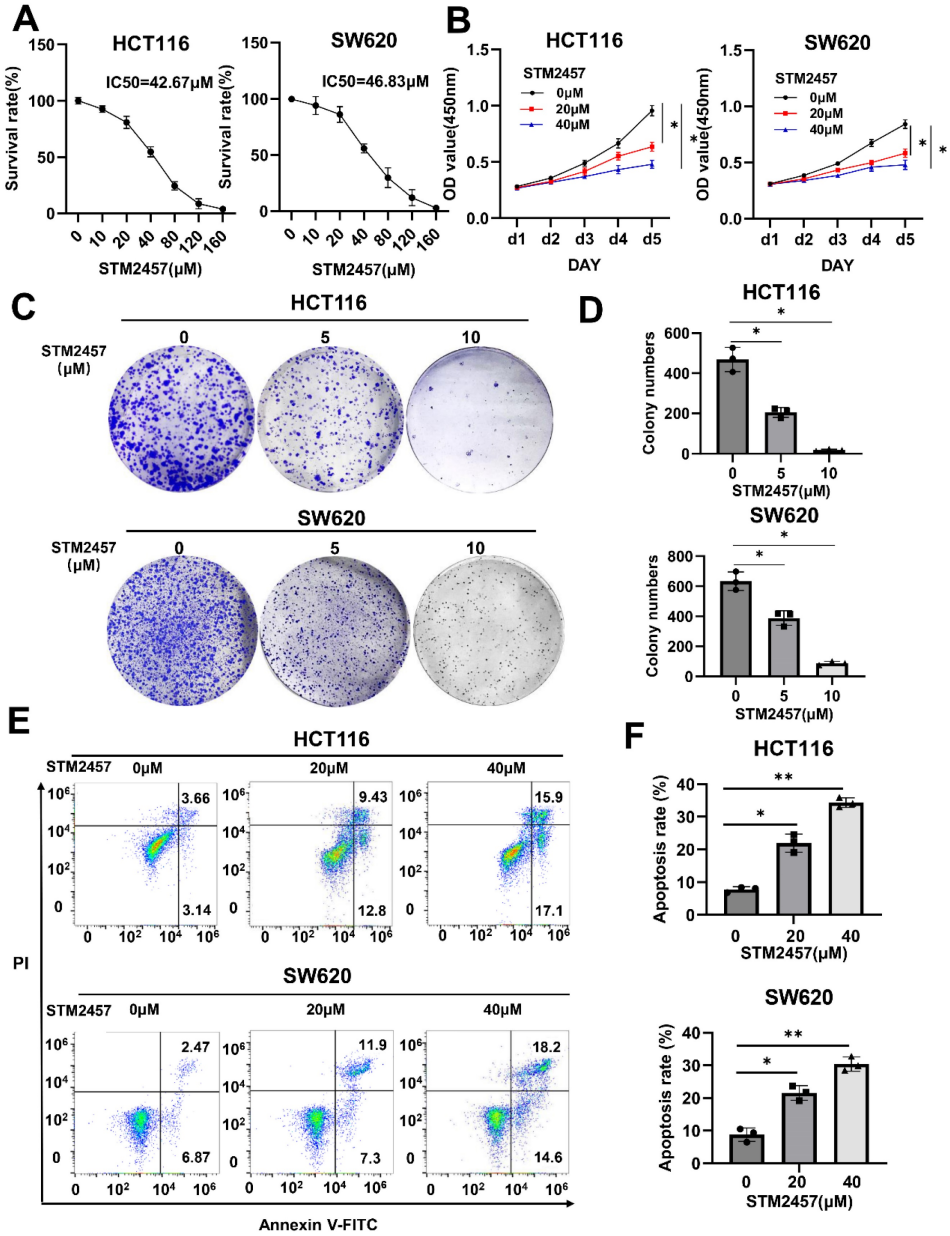
STM2457 inhibits CRC cell progression *in vitro*. (A) After HCT116 and SW620 cells were treated with different concentrations of STM2457 for 3 days, the cell viability was determined to identify the IC50 of STM2457 in CRC. (B) Cell viabilities were explored by CCK-8 for 5 consecutive days when HCT116 and SW620 cells were exposed to STM2457 (0, 20, 40μM). After STM2457 treatment, the proliferation ability of cells decreased. (C) The colony formation ability was detected after being exposed to different concentrations of STM2457 (0, 5, 10μM) for 14 days. In comparison with the control group, STM2457 decreased the number of cell colonies. (D) Flow cytometry was used to demonstrate the proapoptotic ability of STM2457 (0, 20, 40μM). p<0.05 (*) and p<0.01 (**).

The half-maximal inhibitory concentration (IC50) of STM2457 in HCT116 and SW620 cells was measured, respectively (Fig. 1A). After appropriate drug concentrations were selected, CCK-8 assays (Fig. 1B) and colony formation assays (Fig. 1C) were conducted to explore the effect of STM2457 on the proliferation of HCT116 and SW620 cells. The results demonstrated that the cell growth of CRC cells was markedly inhibited by STM2457 in a dose-dependent manner. Subsequently, the impact of STM2457 on the apoptosis of CRC cells was investigated by flow cytometry. As shown in Fig. 1D, the proportion of apoptotic cells increased obviously with incremental concentration of STM2457 in both HCT116 and SW620 cells. These findings suggested that STM2457 can effectively impede proliferation and induce apoptosis of CRC cells* in vitro*.

### STM2457 attenuates the tumorigenicity of CRC cells *in vivo*

**Figure 2:**
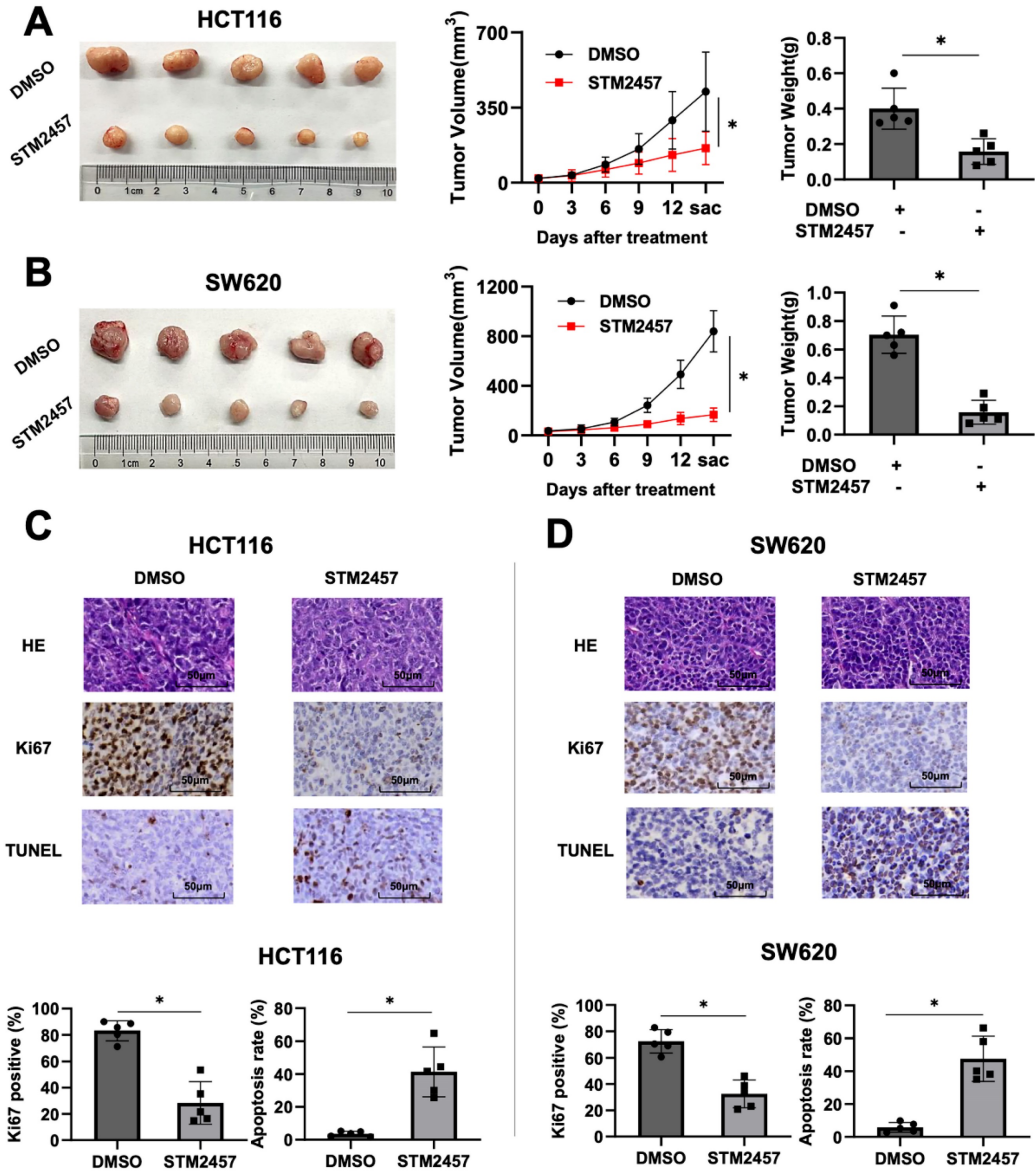
STM2457 attenuated tumor growth of CRC *in vivo*. (A) Representative image of tumor derived from mouse model of subcutaneous xenotransplantation. HCT116 cells were injected subcutaneously into mice and then randomly divided into two groups (n=5). Continuously administered DMSO or STM2457 (50 mg/kg, every three days, intraperitoneal injection) and measured the volume of the tumor for 14 days, then tumors were removed and the weight was measured. (B) The xenograft mouse model derived from SW620 cells was established using the same method as HCT116 cells, and similar results were obtained. (C) and (D) Representative images for H&E and IHC staining for Ki67 and TUNEL analysis and quantification of ki67 positive cells and apoptosis cells in the tumor tissues of mice from different groups. p<0.05 (*).

The xenograft mouse tumor models were established to evaluate the role of STM2457 *in vivo*. Subcutaneous injection was performed using HCT116 and SW620 cells in four-week-old male nude mice (BALB/c). About one week later, we treated mice with DMSO or STM2457 for 14 days and recorded the trend of tumor volume changes during the treatment process. As shown in Fig. 2A, the tumors exposed to STM2457 showed smaller volume, slower growth, and lighter weight compared with the DMSO group. Similarly, consistent results were obtained in the subcutaneous xenograft tumors from SW620 cells (Fig. 2B). Furthermore, H&E and IHC staining was performed on above xenograft tumor tissues. The result showed that Ki67 positive staining was noticeably downregulated in mice group treated with STM2457 in accordance with control group treated with DMSO (Fig. 2C). In addition, the number of apoptotic cells was upregulated illustrated by TUNEL staining assay (Fig. 2D). Based on these data, we concluded that STM2457 has the potential to hinder tumor growth *in vivo*, which is in alignment with the results* in vitro*.

### Identification of ASNS as a downstream effector of STM2457

**Figure 3:**
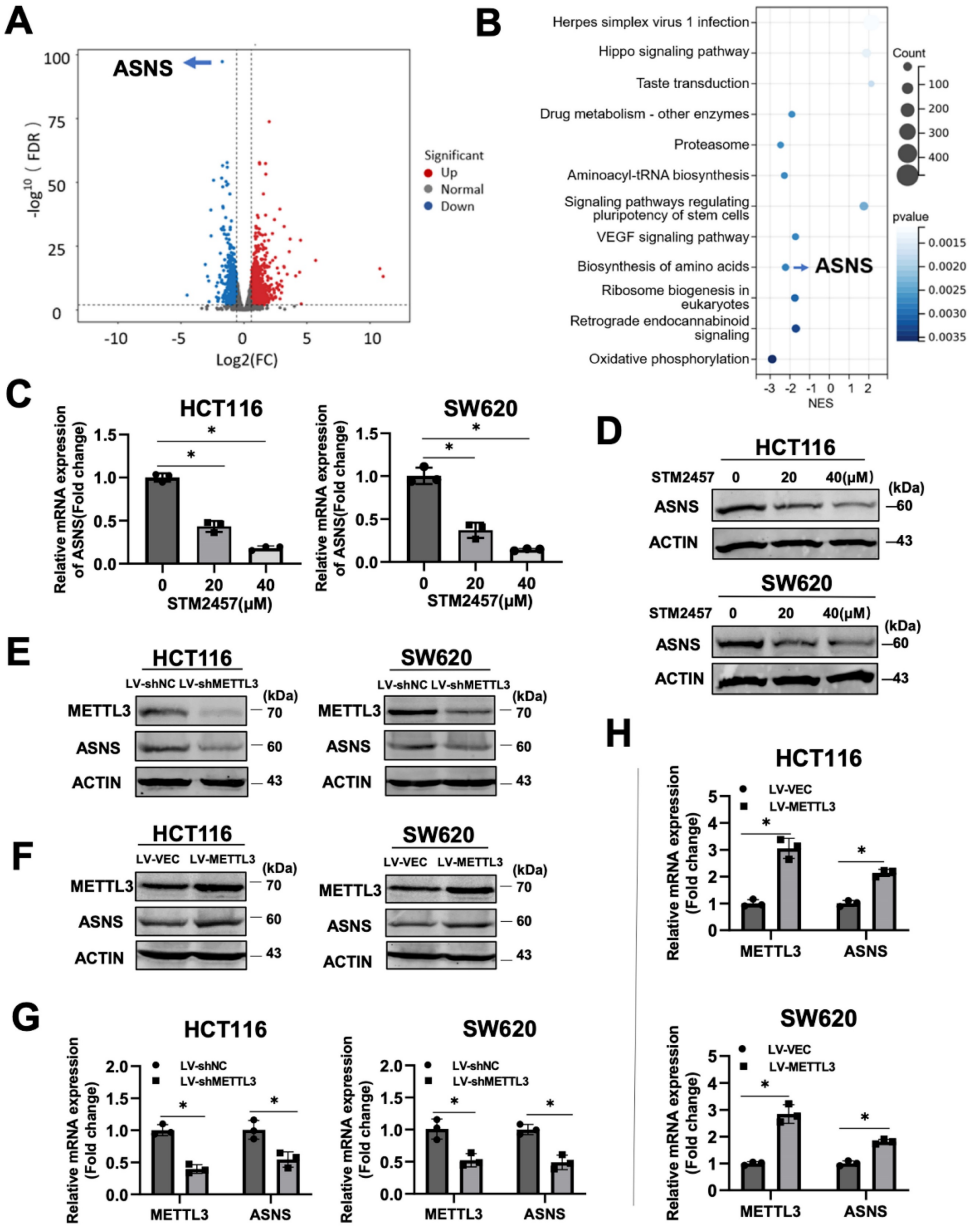
Identification of ASNS as the target gene of STM2457 through RNA-seq. (A) The volcano plot was used to illustrate the differentially expressed genes between two groups (HCT116 cells treated with STM2457 (20 μM) or DMSO for 48 hours). ASNS was the most significantly differentially expressed gene. (B) KEGG analysis on all the DEGs. Among them, ASNS was one of the genes related to amino acid metabolism pathways. (C) RT-qPCR and (D) Western blot confirmed that STM2457 downregulated ASNS levels in CRC cell lines. The expression of ASNS was positively correlated with the dose of the STM2457. (E) Knockdown of METTL3 decreased protein and (G) mRNA expression levels of ASNS. (F) and (H) Overexpression of METTL3 increased expression level of ASNS at protein and mRNA levels as determined by western blot and qPCR approaches. p<0.05 (*).

To investigate the molecular mechanism underlying the anti-cancer activity of STM2457 on CRC cells, we performed RNA sequencing on HCT116 cells treated with STM2457 or DMSO for 48 hours, followed by analysis of differences in global transcriptional expression profiles. In comparison to the control group, there were 2280 differentially expressed genes in the STM2457-treated group, including 1244 upregulated and 1036 downregulated genes. Among them, asparagine synthetase (ASNS) was the most significantly differentially expressed gene (Fig. 3A). Next, Kyoto Encyclopedia of Genes and Genomes (KEGG) analysis on all the differentially expressed genes was performed (Fig. 3B). The results of the KEGG analysis illustrated that the Amino Acid metabolism, Hippo signaling pathway and VEGF signaling pathway were enriched after the treatment with STM2457. To verify the correlation between STM2457 and ASNS in CRC cells, qRT-PCR and western blotting were performed. The results indicated that the mRNA levels of ASNS in HCT116 and SW620 cells were downregulated upon STM2457 treatment in a dose-dependent manner (Fig. 3C-D). On the other hand, we examined whether METTL3 has an effect on ASNS expression since STM2457 is its specific inhibitor. As expected, METTL3 knockdown or overexpression decreased or increased ASNS expression at protein level (Fig. 3E-F) and mRNA level (Fig. 3G-H). Furthermore, we mutated two key sites on METTL3 (D395A and W398A) 43 to make it inactive, and transfected CRC cells with WT-METTL3 with normal enzyme activity and MUT-METTL3 with loss of catalytic activity. The result was as we expected that the overexpression of MUT-METTL3 could not upregulate ASNS compared with WT-METTL3 (Fig. S1A). These results suggested that ASNS was a downstream target of METTL3 and its inhibitor STM2457 in CRC cells.

### STM2457 reduces the expression of ASNS by inhibiting m6A modification

**Figure 4:**
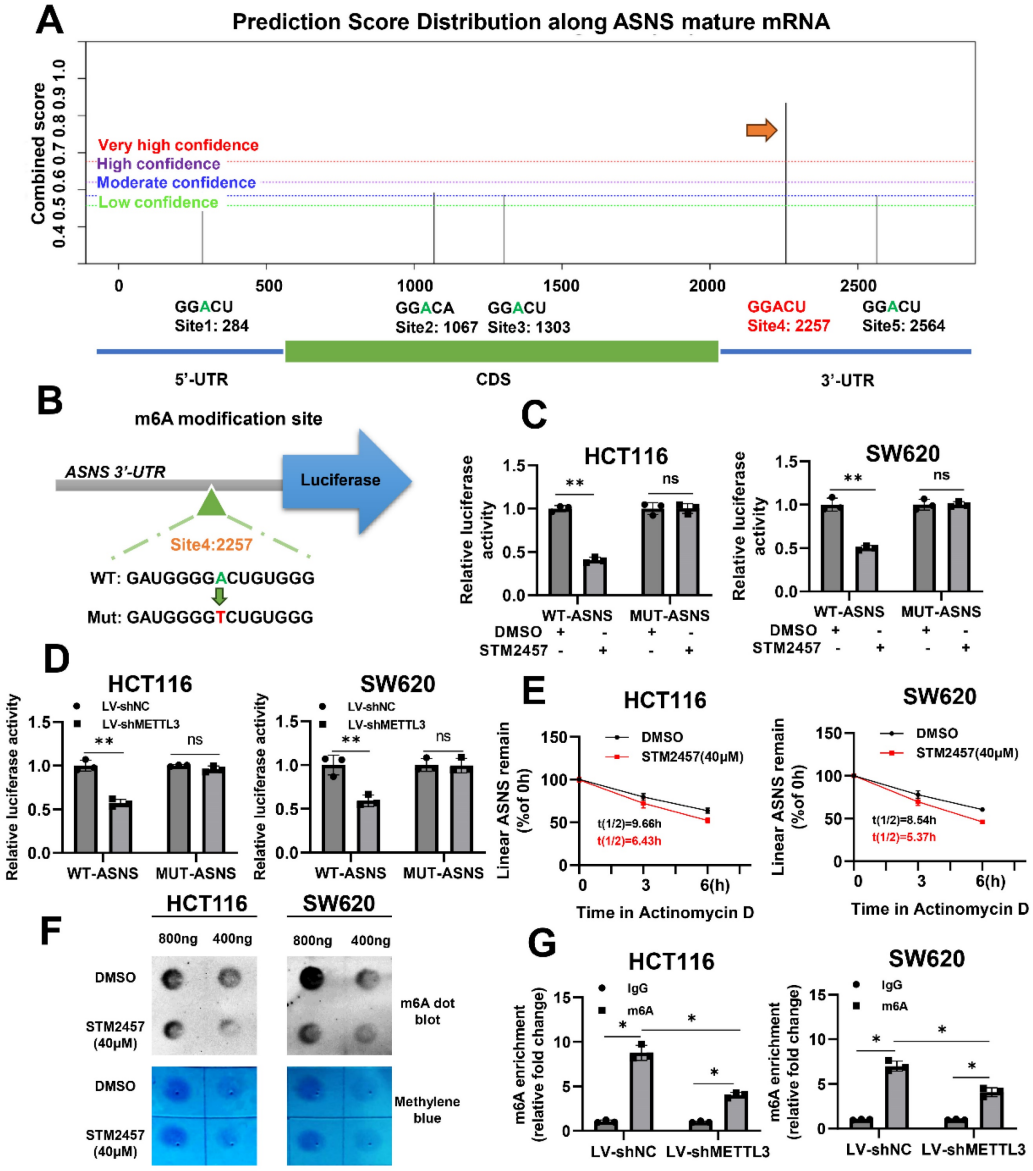
STM2457 reduces the expression of ASNS by inhibiting m6A modification. (A) Predicted distributions of m6A modification sites on ASNS mRNA by SRAMP. The site with the highest confidence was selected for mutation (indicated by the arrow). (B) The pattern map of modification site mutation. (C) The dual luciferase reporter assay confirmed that STM2457 (20 μM) group regulates the activities of WT-ASNS rather than MUT-ASNS in contrast to DMSO group. (D) Knockdown of METTL3 inhibited the activities of WT-ASNS instead of MUT-ASNS. (E) Actinomycin D was added after cells were treated with DMSO or STM2457 for 24 hours and RNA was collected regularly for qRT-PCR to detect the stability of mRNA. (F) RNA m6A dot blotting assay. RNA of the same group was diluted to 800ng/ul and 400ng/ul. The picture (top) is chemiluminescence determination, and the picture (bottom) is methylene blue staining. (G) METTL3 knockdown decreased the binding of m6a antibody with ASNS mRNA in MeRIP-qPCR assays. p<0.05 (*) and p<0.01 (**).

To further investigate if STM2457 downregulates ASNS expression via m6A modification, an m6A site prediction tool SRAMP was be applied to predict the distribution of m6A modification sites on ASNS mRNA and found there were five putative modification sites. One site with particularly high confidence (site 4) was selected for further study (Fig. 4A). Subsequently, the reporter gene plasmids for this site with wild-type or mutant form (A to T) were constructed (Fig. 4B). Dual luciferase reporter gene assay results indicated that STM2457 significantly suppressed wild-type ASNS 3'-UTR activities and had no obvious effect on mutant ASNS 3'-UTR activities (Fig. 4C). The consistent results were also observed when METTL3 was knockdown in the two CRC cell lines (Fig. 4D). For the purpose of verifying the effect of STM2457 on the stability of RNA, actinomycin D was used to test the stability of mRNA. The results showed that the RNA stability of the cells treated with STM2457 was lower than that of the control cells treated with DMSO (Fig. 4E). Furthermore, we carried out the m6A dot blotting assay and found that compared with the control group, the level of m6A in the cells treated with STM2457 was considerably downregulated (Fig. 4F). Concurrently, MeRIP-qPCR assays were employed in METTL3 knockdown cells. The final data revealed that METTL3 silencing reduced the m6A modification of ASNS mRNA (Fig. 4G). These collective findings suggest that STM2457 inhibits ASNS expression through blocking METTL3-mediated m6A modification of ASNS mRNA.

### Overexpression of ASNS can weaken the anti-tumor effect of STM2457

**Figure 5:**
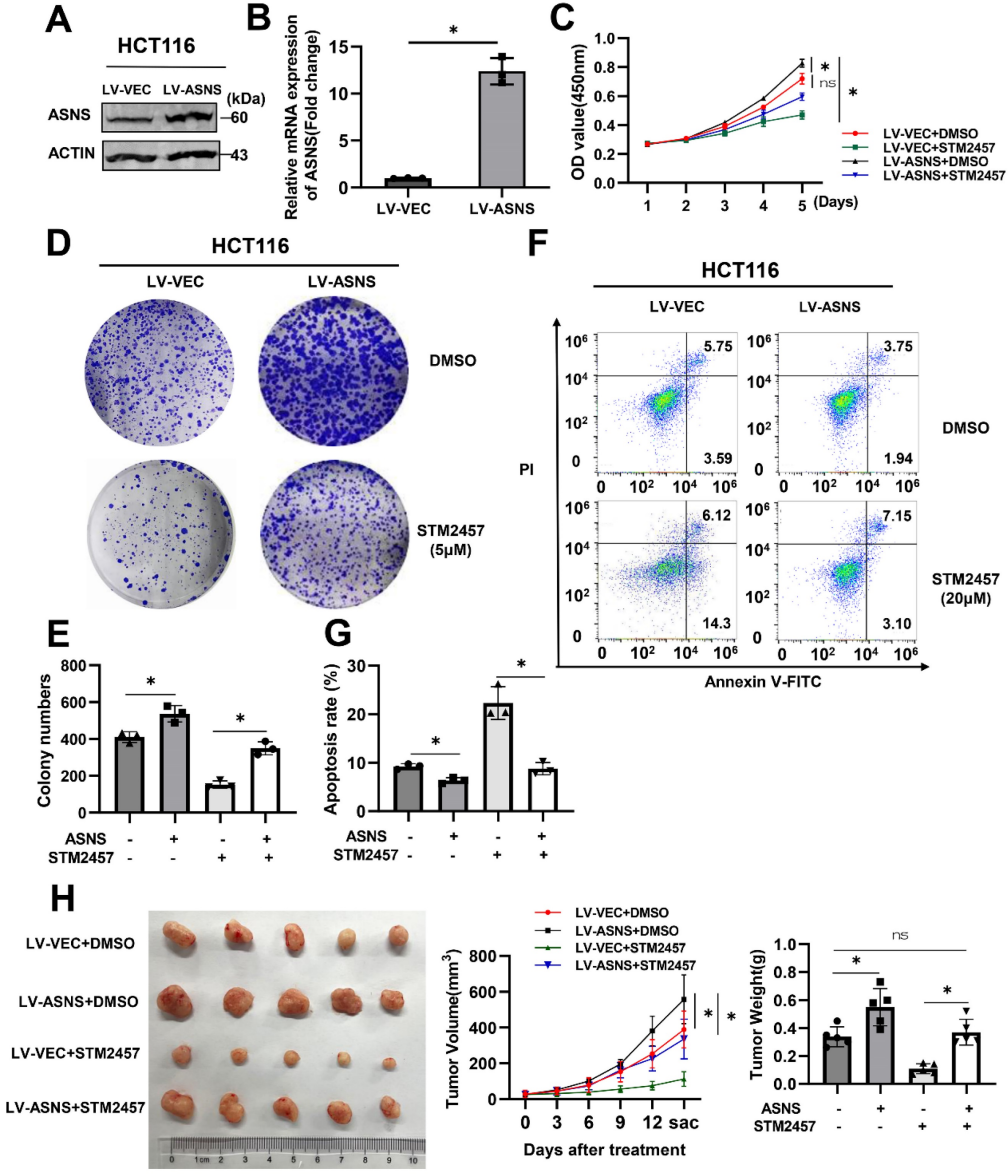
ASNS offsets the anti-tumor effect of STM2457. (A) Western blot and (B) qPCR verified the efficiency of lentivirus-medated ASNS overexpression in HCT116 cells. LV-VEC acts as a negative control. (C) CCK-8 assays were performed in HCT116 cells with or without ASNS overexpression in the presence of STM2457 or in the absence of STM2457. (D) and (E) The cells used as indicated were suffered to colony formation assay. (F) and (G) Apoptosis rates of different treatment groups as indicated were detected by flow cytometry. Overexpression of ASNS can counteract cell apoptosis caused by STM2457. (H) ASNS could offset the anti-tumor effect of STM2457 in a mouse model derived from HCT116 cells as indicated. The tumor picture, volume and weight were shown. p<0.05 (*) and p<0.01 (**)

To clarify whether the inhibitory effect of STM2457 on tumor progression was related to ASNS expression, HCT116 cells were infected with lentivirus to overexpress ASNS and the overexpression efficiency was verified by western blotting (Fig. 5A) and qPCR (Fig. 5B). Thereafter, we performed biological functional assays on the stable ASNS overexpression cell line with the treatment of DMSO or STM2457. The CCK-8 assay showed that ASNS overexpression could apparently attenuate the inhibitory effect caused by STM2457 in HCT116 cells (Fig. 5C). Besides, colony formation assays obtained the analogous results (Fig. 5D-E). Meanwhile, flow cytometry was used to detect apoptosis cells in different treatment groups, and we also found that overexpression of ASNS could reduce the proportion of apoptotic cells induced by STM2457 (Fig. 5F-G). To investigate the existence of these phenomena *in vivo*, xenograft tumor mouse models by subcutaneous injection of control HCT116 cells or ASNS-overexpressed HCT116 cells were constructed. Consistently, the result *in vivo* confirmed that overexpression of ASNS led to a more malignant phenotype in CRC subcutaneous xenograft tumors. Notably, ASNS partially offset the anti-tumor effect of STM2457 in mice overexpressing ASNS along with STM2457 treatment compared to DMSO treatment (Fig. 5H).

Due to the need to consider the potential of STM2457 in clinical treatment, we analyzed the relative CRC clinic data of the downstream target gene ASNS of STM2457. We used TCGA and GEPIA to analyze the correlation of clinical data. Compared with normal tissues adjacent to cancer, ASNS is highly expressed in colorectal cancer tissues of CRC patients (Fig. S2 A). The correlation analysis of ASNS and METTL3 in CRC cancerous tissues showed that there was a positive correlation between them (Fig. S2 B). In addition, we used Kaplan-Meier plotter to analyze the survival of patients with CRC, and the results suggested that patients with high expression of ASNS had a worse prognosis (Fig. S2 C). These results affirmed that ASNS plays a certain oncogene role in CRC.

Based on the above results, it is proposed that STM2457 administration hinders tumor progression via ASNS downregulation, and high expression of ASNS may retard the anti-tumor effect of STM2457 in CRC cells* in vitro* and *in vivo*.

## Discussion

Colorectal cancer has represented a major public health challenge as a consequence of its elevated morbidity and mortality. It is already known that colorectal tumorigenesis is deeply associated with molecular heterogeneity involving genetic, epigenetic, and epitranscriptomic alterations. M6A modification, as the most prevalent post-transcriptional modification of mRNA, can significantly promote the occurrence and development of CRC 25,26. As a result, the research on the m6A regulators is considerably importance. At present, the ethyl ester form of meclofenamic acid (MA), as an inhibitor of FTO 27, has been definitely confirmed to prevent tumor growth. As another inhibitor of FTO, FB23-2 can destroy the proliferation and promote the differentiation of AML cells 28. The first reported inhibitor of METTL3 belonged to nucleoside inhibitors, but they are rarely used because of their poor cell permeability and binding hybridity 36. STM2457, a first-generation bioavailable METTL3 inhibitor, can directly and specifically bind to the METTL3/METTL14 heterodimer and impede METTL3/METTL14 catalytic activity. In 2021, STM2457 has been exploited to reduce engraftment and extend survival* in vivo*, particularly targeting crucial stem cell subpopulations, which shown a therapeutic effect in the pre-clinical models of acute myeloid leukemia (AML) 19. Moreover, in intrahepatic cholangiocarcinoma, inhibiting METTL3 by STM2457 may be an effective approach to arresting malignant tumor growth and overcoming chemotherapy resistance 29. Meanwhile, the anti-neoplasm activity of STM2457 has been explored in lung 20, Medulloblastoma 21 and Osteosarcoma 40. Previous studies on STM2457 have focused on its effect on cell growth activity. In the present study, we explore the biological effects and underlying mechanisms upon STM2457 treatment in CRC cells for the first time.

Our investigations indicated that STM2457 can attenuate the proliferation and promote apoptosis of CRC cells in a dose-dependent manner, which was confirmed in the typical CRC cell lines HCT116 and SW620. Analogous results were obtained from subcutaneous xenograft *in vivo*. We performed high-throughput sequencing of HCT116 cells treated with DMSO or STM2457 to analyze the mechanisms underlying the anti-cancer properties of STM2457. It was shown that ASNS was the most significantly differentially expressed gene associated with the amino acid metabolic pathway. We speculated that STM2457 affected the tumor promotion effect of ASNS by affecting the expression level of ASNS. Subsequent data have confirmed that the mRNA and protein levels of ASNS in colorectal cancer cells treated with STM2457 were significantly downregulated. Notably, the expression level of ASNS was positively correlated with METTL3 in stable cell lines where METTL3 was overexpressed or knocked down. Furthermore, when the catalytic activity of METTL3 was deficient, its overexpression cannot upregulate the expression level of ASNS in CRC.

In other words, the above results verified that STM2457 negatively regulated the expression of ASNS through impeding the catalytic activity of METTL3 in CRC. Since STM2457 was an inhibitor of catalytic activity of METTL3, we predicted the m6A modification site on ASNS and constructed a related mutant plasmid for double luciferase experiment to explore the mechanism. As we expected, both STM2457 administration and METTL3 knockdown hinder the mRNA level of ASNS by diminishing m6A deposition on the specific modification site. We also discussed the negative effect of STM2457 on the mRNA stability of CRC. Consistently, MeRIP assays and m6A dot blotting assays also demonstrated the correlation between ASNS and METTL3 in m6A modification. We reported for the first time that ASNS was intimately related to m6A modification. By means of *in vivo* and *in vitro* assays, we observed that ectopically overexpression of ASNS could partially reverse the STM2457-induced phenotypes of CRC cells. This confirmed that STM2457 hindered the development of CRC through ASNS. Clinical data analysis provided effective evidence for STM2457 as a potential treatment for CRC.

Currently, several studies have shown that asparagine, a non-essential amino acid, can only be produced de novo through the enzymatic synthesis of ASNS 30. This enzyme catalyzes the conversion of aspartate to asparagine via transferring the amide group from glutamine in an ATP-dependent manner 31. Crucially, ASNS has reported to take a critical part in several tumor types because of the asparagine metabolism disorders 32,34. For instance, Maria elucidated a mechanism whereby DON (a glutamine antagonist) could substantially inhibit asparagine production and block the progression of pancreatic ductal adenocarcinoma by inhibiting ASNS 35. Especially in recent years, there have been more and more comprehensive reports on the role of amino acid metabolism in tumors. Some studies have pointed out that amino acid metabolism has a certain influence on tumor drug resistance 37, immunity 38, maintenance of tumor signal transduction 39. For this reason, we hope that the METTL3/ASNS axis can be further studied as a key clue related to immunity or drug resistance in the future. In view of the fact that the animal model used in this paper is relatively single and has some limitations, we can use more animal models in more in-depth research in the future and have a better and more comprehensive report.

In conclusion, our findings highlight that STM2457 has potential to act as a novel therapeutic agent in CRC. STM2457 might be essentially associated with amino acid metabolism pathway via m6A modification of ASNS mRNA. ASNS downregulation caused by STM2457 may mediate the inhibitory effects of STM2457. Our work marked the development of m6A related small molecule inhibitors more feasible.

## Supplementary Material

Supplementary figures.

## Abbreviations

CRC: colorectal cancer
ASNS: asparagine synthetase
m6A: N6- methyladenosine
MeRIP: methylated RNA immunoprecipitation
METTL3: Methyltransferase-like 3
METTL14: Methyltransferase-like 14
MTC: methyltransferase complex
FBS: Fetal bovine serum
DMEM: Dulbecco's Modified Eagle Medium
DMSO: Dimethyl sulfoxide
DEG: Differentially expressed gene. GEPIA: Gene Expression Profiling Interactive Analysis
KEGG: Kyoto Encyclopedia of Genes and Genomes
RFS: Recurrence free survival
TCGA: Gene Expression Profiling Interactive Analysis
OS: Overall survival
