# Supplementary material for: METTL3 Inhibition Suppresses Cell Growth and Survival in Colorectal Cancer via ASNS Downregulation
Source: J Cancer. 2024 Jul 16;15(15):4853–65. doi: 10.7150/jca.96760 (PMC11310885; doi:10.7150/jca.96760)
Supplement: Supplementary file 1 — Supplementary figures. [file jcav15p4853s1.pdf]

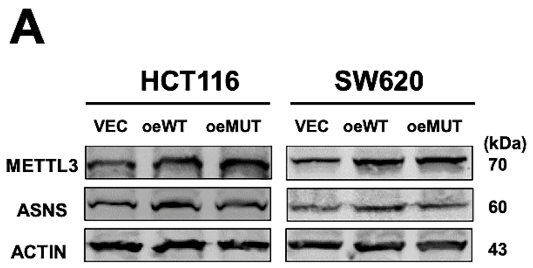

Fig.S1 The effect of lack of catalytic activity of METTL3 on the expression of ASNS. (A) The overexpression of wild-type METTL3 could upregulate ASNS rather than the mutation-type of losing the catalytic function.

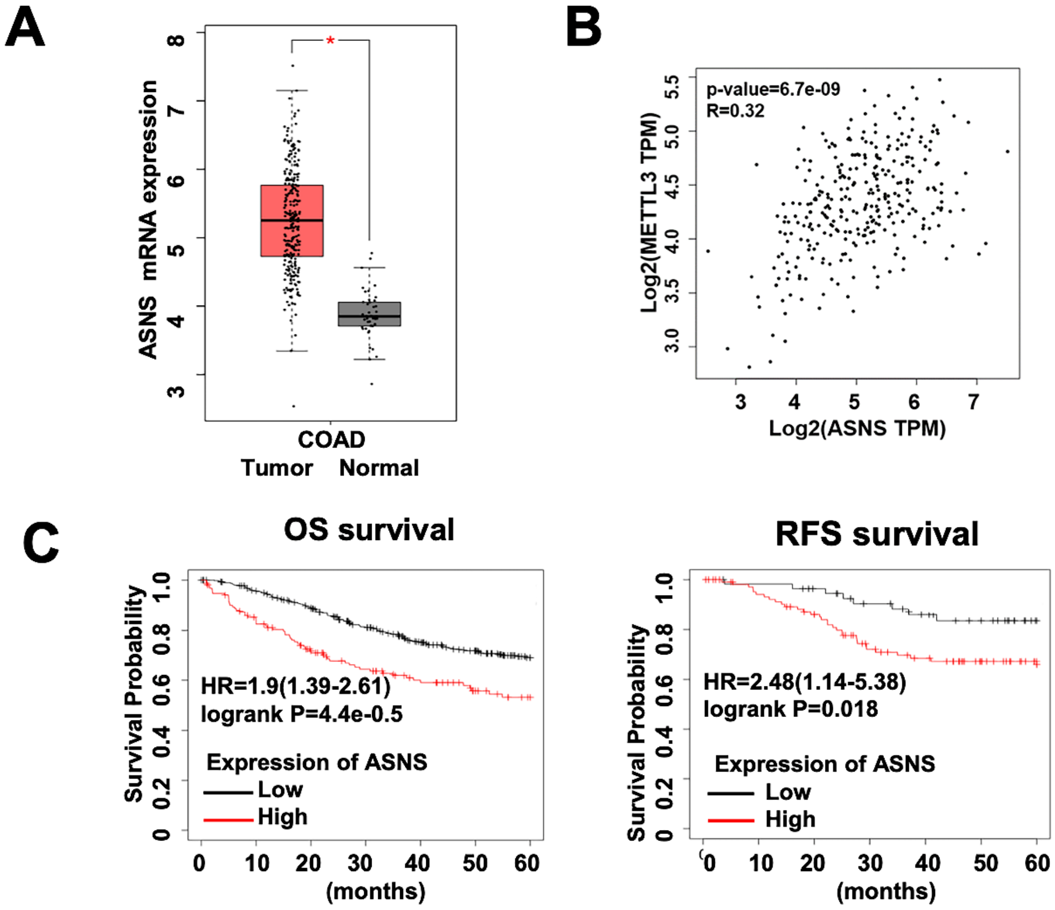

Fig.S2 The expression of ASNS and its correlation with prognosis in CRC. (A) ASNS mRNA expression of COAD database of GEPIA. (B) Correlation analysis between ASNS and METTL3 in COAD database of GEPIA. (C) Overall survival (OS) and Recurrence free survival (RFS) of COAD data set (GSE17538 n=232) in Kaplan-Meier plotter.
